# Photoluminescent lead(II) coordination polymers stabilised by bifunctional organoarsonate ligands

**DOI:** 10.1088/1468-6996/16/2/024803

**Published:** 2015-03-17

**Authors:** Jian-Di Lin, Camelia I Onet, Wolfgang Schmitt

**Affiliations:** 1 Department of Applied Chemistry, College of Life Sciences, Fujian Agriculture and Forestry University, Fuzhou, Fujian 350002, People’s Republic of China; 2 School of Chemistry & CRANN, University of Dublin, Trinity College, Dublin 2, Ireland

**Keywords:** coordination polymers, metal-organic frameworks, lead, phtoluminesence, x-ray crystallography

## Abstract

Four lead(II) coordination polymers were isolated under hydro(solvo)thermal conditions. The applied synthetic methodology takes advantage of the coordination behaviour of a new bifunctional organoarsonate ligand, 4-(1, 2, 4-triazol-4-yl)phenylarsonic acid (H_2_TPAA) and involves the variation of lead(II) reactants, metal/ligand mole ratios, and solvents. The constitutional composition of the four lead(II) coordination polymers can be formulated as [Pb_2_(TPAA)(HTPAA)(NO_3_)]·6H_2_O (**1**), [Pb_2_(TPAA)(HTPAA)_2_]·DMF·0.5H_2_O (DMF = N, N-Dimethylformamide) (**2**), [Pb_2_Cl_2_(TPAA)H_2_O] (**3**), and [Pb_3_Cl(TPAA)(HTPAA)_2_H_2_O]Cl (**4**). The compounds were characterized by single-crystal and powder x-ray diffraction techniques, thermogravimetric analyses, infra-red spectroscopy, and elemental analyses. Single-crystal x-ray diffraction reveals that **1** and **2** represent two-dimensional (2D) layered structures whilst **3** and **4** form three-dimensional (3D) frameworks. The structures of **1**, **2,** and **4** contain one-dimensional (1D) {Pb^II^/AsO_3_} substructures, while **3** is composed of 2D {Pb^II^/AsO_3_} arrays. Besides their interesting topologies, **1**–**4** all exhibit photoluminescence properties in the solid state at room temperature.

## 1. Introduction

Over the past decades, the design and synthesis of coordination polymers (CPs) developed into an active research area due to the interesting physicochemical properties and potential applications of the materials in electro-optical devices, in catalysis, sensors, gas storage, or separation materials [1–11]. The hydro(solvo)thermal techniques provide powerful synthetic methodologies for the construction of CPs through the self-assembly of metal ions and designed organic ligands involving elevated temperatures and pressures. However, up until now, it still remains a considerable challenge to achieve controllable preparation conditions to produce crystalline CP materials with desired topological and chemical attributes. The reason for this stems from the complex formation conditions that influence the self-assembly and crystallization process; the applied synthetic approach is influenced simultaneously by numerous parameters, including the structural characteristics of the organic ligands, the coordination geometry of central metal ions, the metal/ligand molar ratio, available counteranions, the solvent system, the pH value of the solution, temperature, pressure, and reaction time [12–16]. Importantly, the nature of the organic ligand often influences the topology, the stability, the formation, and the crystallization conditions of the resulting hybrid materials.

The coordination chemistry of metal phosphonates has received significant attention over the last decades due to a number of industrial foreseeable applications of the compounds [17, 18]; phosphonates have been used to prepare zero-dimensional molecular species and coordination polymers [19–25]. To date, diverse bi-functional organophosphonic acid ligands containing various functional auxiliary groups (crown ether, amine, hydroxyl, and/or carboxylate, etc) have been used to control the formation and properties of CP [26–33]. For example, recently a bifunctional aromatic organophosphonic acid ligand that contains a triazole moiety, 4-(1, 2, 4-triazol-4-yl)phenylphosphonic acid (H_2_ptz), has been synthesized and was used to stabilize three two-dimensional (2D) CPs containing Ni(II), Co(II), or Mn(II) metal ions [34, 35]. Up until now, most of the reported extended metal phosphonates display layered structures, while one-dimensional (1D) and porous three-dimensional (3D) networks have rarely been reported [36–40].

Organoarsonic acids (R-AsO_3_H_2_) display geometrical characteristics that closely relate to those of analogous phosphonic acids (R-PO_3_H_2_); thus metal arsonates are expected to show structural and topological attributes that are similar to those of the metal phosphonates. However, the larger As(V) ionic radius and longer As−O bonds can be expected to result in modified architectures and distinguishing features to the corresponding phosphonate system. Pronounced differences of the pK_a_ values may further distinguish the coordination chemistry of arsonates and phosphonates. So far, reports on metal arsonates are rather scarce. Most of the reported metal organoarsonates are hybrid polyoxometalate (POM) clusters based on V, Mo, or W [41–52]. In particular, Zubieta’s research group has extensively explored the formation of such hybrid POMs [45, 47, 48, 50, 51]. We have reported several capsular arsonate-stabilized polyoxovanadates that incorporate substituted R-phenylarsonate and investigated their 3D assembly [53–56]. In such POMs, each arsonate functionality bridges several metal centers and shows a coordination behaviour that is closely comparable to that of the corresponding phosphonate-stabilized POMs [33, 53–56]. Synthetic approaches to Sn- and Pd-based organoarsonate complexes have also been explored under hydro(solvo)thermal conditions [57–59]. Recently, Tian *et al* reported examples of uranyl arsonates that are stabilized by phenylarsonate ligands [60]. To date, arsonate-stabilized metal-organic CPs are significantly less developed and investigated compared to the corresponding phosphonate compounds. The reported CPs predominantly contain *s*-, *d*-, and *f*-block metal ions, while significantly less attention has been paid to the *p*-block-based metal ions. This is somewhat surprising, as these types of CPs lend themselves to important applications in electroluminescent and photovoltaic conversion devices or fluorescent sensors [61–67]. As a heavy *p*-block metal ion, lead(II) may provide a potential opportunity to construct novel extended inorganic hybrids with fascinating topologies and interesting optical properties. Its large radius, variable stereochemical activity, and flexible coordination environment make a Pb(II) system to be an interesting candidate to prepare organoarsonate-stabilized network structures. To date, only very few lead arsonates have been reported in the literature [68, 69].

Here we report the synthesis and characterization of 4 lead(II)-organoarsonate CPs that were obtained under solvothermal reaction conditions using a bifunctional arsonic acid ligand, 4-(1, 2, 4-triazol-4-yl)phenylarsonic acid (H_2_TPAA). Our synthetic approach uses different lead(II) reactants that were employed at different metal/ligand mole ratios in solvents of different polarity and resulted in [Pb_2_(TPAA)(HTPAA)(NO_3_)]·6H_2_O (**1**), [Pb_2_(TPAA)(HTPAA)_2_]·DMF·0.5H_2_O (**2**), [Pb_2_Cl_2_(TPAA)H_2_O] (**3**), and [Pb_3_Cl(TPAA)(HTPAA)_2_H_2_O]Cl (**4**). Among these four coordination polymers, **1** and **2** represent 2D layered structures whilst **3** and **4** form 3D framework structures. To the best of our knowledge, the reported arsonate coordination polymers mainly adopt 1D chain and 2D layer structures [70–74], whilst 3D dimensional structures are very rare [75, 76]. The structures of **1**, **2**, and **4** contain 1D {Pb-AsO_3_} structural motifs, while **3** is composed of 2D {Pb-AsO_3_} substructures. The luminescence properties of **1**–**4** have been investigated in the solid state at room temperature.

## 2. Experimental section

### 2.1. Materials and instrumentation

The H_2_TPAA ligand was synthesized from *N*, *N*-dimethylformamide azine dihydrochloride using a general procedure previously reported by us [77, 78]. All reagents were purchased from Sigma-Aldrich and used as received without further purification. ^1^H and ^13^C nuclear magnetic resonance data were recorded on a Bruker DPX 400 spectrometer (400.13 MHz for ^1^H, 100.63 MHz for ^13^C). Fourier transform infrared spectroscopy (FTIR) data were collected on a PerkinElmer Spectrum One FTIR Spectrometer. Thermogravimetric analyses (TGAs) were performed in air on a Perkin Elmer Pyrus 1 TGA from 30–800 °C at a heating rate of 10° C/min. Powder x-ray diffraction (PXRD) data were recorded on a Siemens D500 x-ray diffractometer at 40 kV, 30 mA with Cu-K*α* radiation (*λ* = 1.54 056 Å), with a scan speed of 3°/min and a step size of 0.05° in 2*θ* at room temperature. The simulated patterns were derived from the Mercury Version 1.4 software (http://www.ccdc.cam.ac.uk/products/mercury/) using the data obtained from the single-crystal x-ray diffraction experiments (cif files). Elemental analyses (C, H, and N) were obtained from the Microanalysis Laboratory, School of Chemistry and Chemical Biology, University College Dublin. Solid state fluorescence measurements were carried out using a Fluorolog®-3 Spectrofluorometer.

### 2.2. Syntheses of the complexes

**[Pb**_**2**_**(TPAA)(HTPAA)(NO**_**3**_**)]·6H**_**2**_**O** (**1**) Pb(NO_3_)_2_ (0.1 mmol), H_2_TPAA (0.1 mmol), and 5 mL of H_2_O were added into a 10 mL reaction vial and sealed. The mixture was heated to 100 °C and kept at this temperature for 1 d. Colourless needle crystals of **1** were obtained and washed with H_2_O before drying in air at room temperature (yield: 31 mg, 55% based on Pb(NO_3_)_2_). Elemental analysis (%): calculated For. C_16_H_25_N_7_O_15_Pb_2_As_2_ (1119.65), C 17.16 H 2.25 N 8.76; Found C 17.23 H 2.50 N 8.71. IR (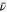/cm^−1^): 3076 (w), 1739 (m), 1632 (w), 1597 (m), 1521 (s), 1419 (w), 1374 (s), 1316 (s), 1244 (s), 1218 (s), 1098 (s), 1039 (vw), 1022 (vw), 1003 (m), 963 (vw), 864 (m), 851 (m), 792 (vs), 733 (w), 713 (w).

**[Pb**_**2**_**(TPAA)(HTPAA)**_**2**_**]·DMF·0.5H**_**2**_**O** (**2**) PbBr_2_ (0.1 mmol), H_2_TPAA (0.15 mmol), and 5 mL DMF (DMF = N, N-Dimethylformamide) were added into a 10 mL reaction vial and sealed. The mixture was heated to 100 °C and kept at this temperature for 4 d. Colourless needle crystals of **2** were obtained and washed with DMF and ethanol before drying in air at room temperature (yield: 45 mg, 69% based on PbBr_2_). Elemental analysis (%): calculated For. C_27_H_25_As_3_N_10_O_10.5_Pb_2_ (1296.71), C 25.01 H 1.94 N 10.80; Found C 24.90 H 2.05 N 10.75. IR (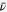/cm^−1^): 3117 (w), 2918 (w), 1664 (s), 1597 (m), 1524 (s), 1413 (vw), 1372 (w), 1327 (w), 1294 (vw), 1245 (s), 1100 (s), 1006 (w), 995 (m), 956 (w), 873 (m), 811 (vs), 727 (m), 708 (m), 670 (vw), 659 (vw).

**[Pb**_**2**_**Cl**_**2**_**(TPAA)H**_**2**_**O]** (**3**) PbCl_2_ (0.2 mmol), H_2_TPAA (0.1 mmol), and 5 mL of H_2_O were added into a 10 mL reaction vial and sealed. The mixture was heated to 100 °C and kept at this temperature for 1 d. Colourless prism crystals of **3** were obtained and washed with H_2_O before drying in air at room temperature (yield: 59 mg, 55% based on PbCl_2_). Elemental analysis (%): calculated For. C_8_H_8_AsCl_2_N_3_O_4_Pb_2_ (770.37), C 12.47 H 1.05 N 8.45; Found C 12.53 H 1.13 N 8.39. IR (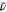/cm^−1^): 3373 (w), 3111 (w), 1614 (w), 1592 (w), 1518 (s), 1414 (m), 1368 (vw), 1326 (w), 1291 (w), 1262 (vw), 1241 (s), 1196 (vw), 1097 (s), 1086 (m), 1021 (w), 1006 (m), 964 (vw), 859 (s), 835 (m), 816 (s), 782 (vs), 725 (m), 666 (vw).

**[Pb**_**3**_**Cl(TPAA)(HTPAA)**_**2**_**H**_**2**_**O]Cl** (**4**) PbCl_2_ (0.1 mmol), H_2_TPAA (0.2 mmol), and 5 mL of H_2_O were added into a 10 mL reaction vial and sealed. The mixture was heated to 100 °C and kept at this temperature for 1 d. Colourless needle crystals of **4** were obtained and washed with H_2_O before drying in air at room temperature (yield: 27 mg, 54% based on PbCl_2_). Elemental analysis (%): calculated For. C_24_H_22_As_3_Cl_2_N_9_O_10_Pb_3_ (1513.74), C 19.04 H 1.46 N 8.33; Found C 19.9 H 1.52 N 8.29. IR (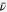/cm^−1^): 3091 (w), 2213 (vw), 1697 (vw), 1598 (m), 1581 (vw), 1530 (s), 1470 (vw), 1415 (w), 1373 (w), 1322 (w), 1293 (vw), 1267 (vw), 1249 (s), 1195 (vw), 1167 (vw), 1099 (s), 1026 (w), 1012 (vw), 1001 (m), 958 (w), 884 (w), 864 (m), 844 (w), 823 (s), 804 (s), 770 (s), 746 (s), 722 (m), 705 (vw).

### 2.3. X-ray crystallography

The data collections of **1** and **2** were carried out on a Bruker APEX Duo CCD x-ray diffractometer at 100 K using graphite-monochromated Cu-K*α* (*λ* = 1.54 178 Å) and Mo-K*α* radiation (*λ* = 0.71 073 Å), respectively, while those of **3** and **4** were carried out on a Rigaku 724 CCD x-ray diffractometer using graphite-monochromated Mo-K*α* radiation (*λ* = 0.71 073 Å). The data collection temperatures of **1**–**4** were 100, 123, 293, and 95 K, respectively. The structures of these four coordination compounds were solved by direct methods using SHELXS-97, integrated using the OLEX2 software [79] and refined with full-matrix least squares on *F*^2^ using the SHELXL-97 program [80]. All the non-hydrogen atoms were refined anisotropically. All the hydrogen atoms of **1** and **3** were refined on calculated positions. For **2** and **4**, the hydrogen atoms belonging to As1O_3_ groups (H13 and H33 in **2**, H13 and H23 in **4**) were located from their respective difference maps; the other hydrogen atoms in **2** and **4** were all refined on calculated positions. The DMF guest molecule exhibits a positional disorder over two sites (C43, O41 and C43′, O41′). The site occupancies of C43, O41, C43′, O41′ are all 0.5. Details of x-ray analysis, including the crystal parameters, data collection, and refinement parameters for compounds **1**–**4** are summarized in table 1. Selected bond lengths, angles, and hydrogen-bond interactions are summarized in tables S1.1–S4.2 (SI). Further details of the crystal structure determination have been deposited at the Cambridge Crystallographic Data Centre (CCDC). CCDC numbers for **1**–**4**: 999 421–999 424, respectively.

**Table 1.** Crystal data and structure refinement information for compounds **1**–**4**.

Compound	1	2	3	4
Empirical formula	C_16_H_25_N_7_O_15_Pb_2_As_2_	C_27_H_25_As_3_N_10_O_10.5_Pb_2_	C_8_H_8_AsCl_2_N_3_O_4_Pb_2_	C_24_H_22_As_3_Cl_2_N_9_O_10_Pb_3_
Formula weight	1119.65	1296.71	770.37	1513.74
Crystal system	monoclinic	triclinic	orthorhombic	triclinic
Space group	*C*2*/c*	*P-*1	*Pccn*	*P-*1
a/Å	37.369(2)	7.2735(3)	24.584(5)	7.2305(14)
b/Å	7.2157(4)	15.8672(7)	7.4927(15)	13.708(3)
c/Å	27.4536(16)	16.0766(7)	17.155(3)	17.480(4)
*α*/°	90.00	108.6790(10)	90.00	79.37(3)
*β*/°	130.137(2)	93.1710(10)	90.00	83.26(3)
*γ*/°	90.00	94.5260(10)	90.00	81.70(3)
V/Å^3^	5659.4(6)	1745.80(13)	3160.0(11)	1677.5(6)
Z	8	2	8	2
Temperature/K	100	123	293	95
*D*_calcd_ (g/cm^3^)	2.628	2.467	3.239	2.997
*μ*/mm^-1^	26.208	12.524	23.712	18.183
Reflections collected	132 14	303 29	132 89	196 50
Independent reflections	4137	6452	2933	5906
*R*_*int*_	0.0331	0.0177	0.0731	0.0376
*R*_1_^a^, *wR*_2_^b^ [I > 2*σ*(I)]	0.0329, 0.0838	0.0172, 0.0379	0.0521, 0.1278	0.0392, 0.0983
*R*_1_^a^, *wR*_2_^b^ (all data)	0.0341, 0.0847	0.0188, 0.0386	0.0544, 0.1297	0.0397, 0.0987
Goodness-of-fit	1.103	1.049	1.096	1.138

a *R*_1_ = *∑*[|*F*_*o*_|- |*F*_*c*_|]/*∑*|*F*_*o*_|.

b *wR*_2_ = [*∑w*(*F*_*o*_^2^- *F*_*c*_^2^)^2^/*∑w*(*F*_*o*_^2^)^2^]^0.5^.

## 3. Results and discussion

### 3.1. Syntheses

Compounds **1**–**4** were reproducibly obtained in moderate yields under hydro(solvo)thermal conditions upon combination of lead salts and H_2_TPAA in H_2_O or DMF. The products form depending on the nature of the metal salt, the relative metal:ligand mole ratios, or the solvent system. Compound **1** selectively forms when Pb(NO_3_)_2_ and H_2_TPAA are reacted in H_2_O. When the nitrate reactant is substituted by PbCl_2_, compounds **3** and **4** are obtained, depending on the relative metal:ligand mole ratios. Compound **3** forms when equimolar ratios of PbCl_2_ and H_2_TPAA are reacted with each other in H_2_O. Higher yields can be obtained when a PbCl_2_:H_2_TPAA mole ratio of 2:1 is used. However, when the relative quantity of PbCl_2_ in the reaction system is reduced to a mole ratio of 1:2, phase-pure crystals of compound **4** are obtained. Reactions of PbCl_2_ and H_2_TPAA at a mole ratio of 1:3 lead to product mixtures of **4** and a white unidentified precipitate. Corresponding hydrothermal reactions of PbX_2_ (X = OAc or F) and H_2_TPAA in water did not lead to phase-pure products or crystals suitable for single-crystal x-ray diffraction. Compound **2** requires PbBr_2_ as a starting material and forms in DMF. Best yields are obtained when the metal salt and the ligand are reacted at 100 °C for 4 d at stoichimetric ratios as given by the formula of **2**. 1:1 mole ratios or reduced reaction time lead to the co-precipitation of a white unidentified product. If the reactions of PbX_2_ (X = Cl, OAc, or F) and H_2_TPAA were carried out in a DMF solvent system, then either a colourless clear solution (for Pb(OAc)_2_) or floccule/microcrystalline solids (for PbF_2_ or PbCl_2_) are obtained.

### 3.2. Structural description of the complexes

**Figure 1.**
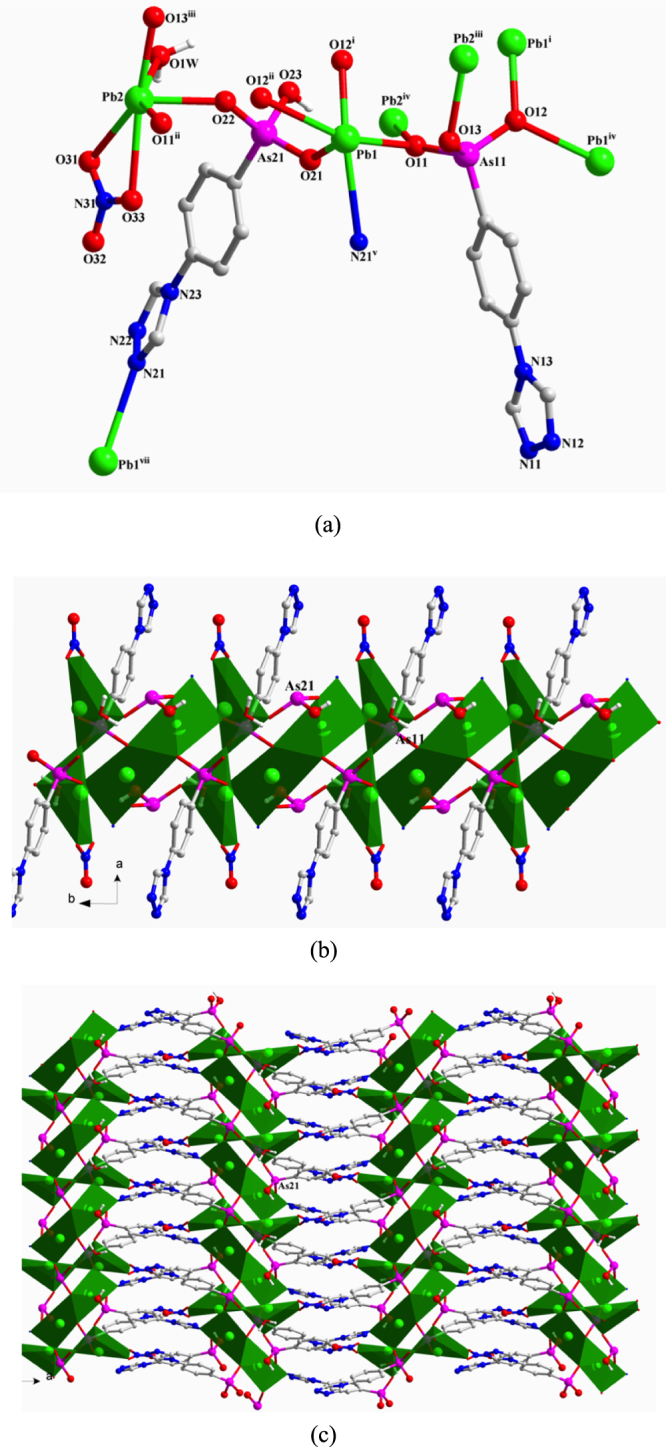
(a) The coordination environment of Pb(II) ions and the coordination modes of ligands in **1**. Symmetry codes: (i) −*x*, −*y* + 1, −*z*; (ii) *x*, *y* + 1, *z*; (iii) −*x*, −*y* + 2, −*z*; (iv) *x*, *y*−1, *z*; (v) −*x* + 1/2, *y*−1/2, −*z* + 1/2; (vii) −*x* + 1/2, *y* + 1/2, −*z* + 1/2. (b) Polyhedral representation of the 1D substructure comprising Pb(II) ions, the two different AsO_3_ functionalities, and the NO_3_^–^ anion in **1**. (c) Polyhedral representation of the 2D layer structure of **1**.

**[Pb**_**2**_**(TPAA)(HTPAA)(NO**_**3**_**)]·6H**_**2**_**O** (**1**) Compound **1** crystallizes in the monoclinic space group *C*2*/c*. The asymmetric unit consists of two Pb(II) ions, one fully deprotonated TPAA^2–^ ligand, one HTPAA^–^ anion (denoted as L_As11_ and L_As21_), one NO_3_^–^ anion, one coordinated water molecule, and five lattice water molecules (the occupancies of O6W and O7W are both 0.5) (SI, figure S1(a)). Both two Pb(II) ions are in hemidirected coordination spheres. The Pb1(II) ion is five-coordinate with four arsonate oxygen atoms and one nitrogen atom deriving from five different ligands. The Pb2(II) ion is six-coordinate, whereby three arsonate oxygen atoms originate from three different ligands, two oxygen atoms from a NO_3_^–^ anion and one water molecule. The Pb–O distances (2.331(5)–2.706(4) Å) are close to those reported for other lead(II) organo arsonates [68, 69]. The long distance of the Pb1–N21^v^ bond (2.8372 (77) Å) is comparable to some corresponding long Pb–N bonds reported in the literature [81–83]. The As–O distances of the {As11O_3_} group are 1.684(5), 1.686(5), and 1.687(5) and of {As21–O} are 1.666(5), 1.667(5), and 1.735(5) Å. Considering the charge balance of **1**, and the fact that the As21–O23 (1.735(5) Å) bond is the longest among these As–O bonds, O23 was assigned as a hydroxyl oxygen atom. According to the research of Shimoni-Livny *et al* [84], the repulsion effect of the 6s^2^ lone pair electrons may result in long bonds and weak coordinative binding interactions involving Pb(II) ions. In light of these results, we have used a bond order calculation [85] to further characterize the coordination environment of the Pb(II) center in **1**. The valence of Pb1 associated with the four Pb–O bonds is 1.675. Further calculations including the weak Pb1–N21^v^ interaction increase the valence of Pb1 from 1.675 to 1.864; the valence of Pb2 is 1.907, which is calculated considering all the six Pb2–O bonds (the calculated valence contribution of the long Pb2–O31 interaction (2.8821(49)) Å is 0.1248). L_As11_ and L_As21_ adopt (*κ*O11, O11**-***κ*O12, O12-*κ*O13)-*μ*_5_ and (*κ*O21**-***κ*O22-*κ*N21)-*μ*_3_ coordination modes, respectively (figure 1(a)). The two different {AsO_3_} functionalities of L_As11_ and L_As21_ link the Pb(II) ions in **1** into 1D chains that extend in the direction of the crystallographic *c*-axis (figure 1(b)) and are connected to each other by a L_As21_ spacer to form a 2D layer structure (figure 1(c)). Further, parallel aligned 2D layers stack in the direction of the crystallographic *b*-axis in an *ABC* fashion to form a 3D supramolecular architecture through hydrogen-bonds involving the H-bond donor/acceptor pairs O2W—H2WA···N22^v^ and O7W—H7WB···N11^vii^ [Symmetry codes: (v) –*x* + 1/2, *y*−1/2, −*z* + 1/2; (vii) −*x* + 1/2, *y* + 1/2, −*z* + 1/2]. Strong hydrogen-bond interactions also occur between the water molecules (SI, figure S1(b)).

**Figure 2.**
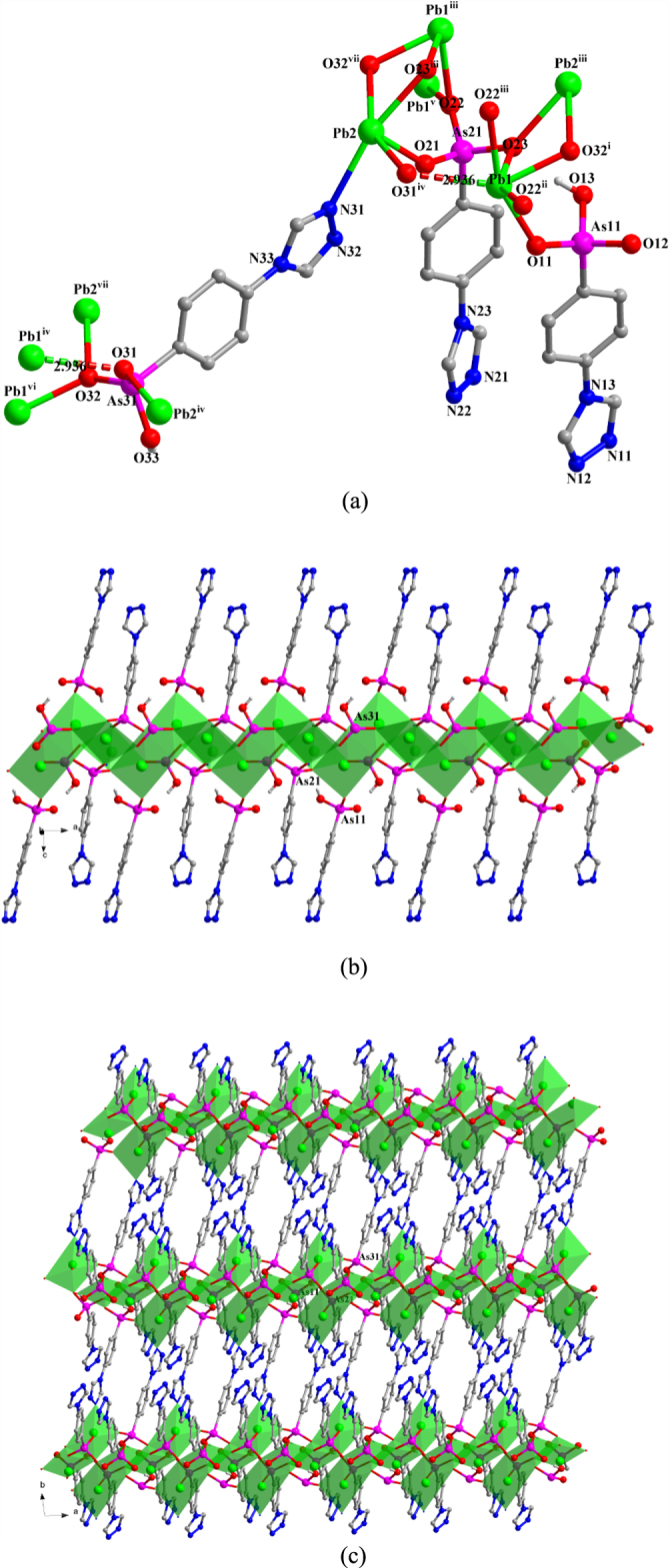
(a) The coordination environment of the Pb(II) ions and the coordination modes of the ligands in **2**. Symmetry codes: (i) *x*, *y*−1, *z*; (ii) *x* + 1, *y*, *z*; (iii) −*x*, −*y*, −*z* + 1; (iv) −*x* + 1, −*y* + 1, −*z* + 1; (v) *x*−1, *y*, *z*; (vi) *x*, *y* + 1, *z*; (vii) –x, 1-y, 1-z. (b) Polyhedral representation of the one-dimensional sub-structure in **2** comprising of Pb(II) ions and the three different {AsO_3_} functionalities (view in the direction of the crystallographic *b*-axis). (c) Polyhedral representation of the two-dimensional structure in **2** (view in the direction of the crystallographic *b*-axis).

**[Pb**_**2**_**(TPAA)(HTPAA)**_**2**_**]·DMF·0.5H**_**2**_**O** (**2**) Single-crystal x-ray diffraction measurements reveal that compound **2** crystallizes in the triclinic space group *P-*1. The asymmetric unit of **2** consists of two Pb(II) ions, one fully deprotonated TPAA^2–^ ligand, two HTPAA^–^ anions (denoted as L_As11_, L_As21_, and L_As31_), one lattice DMF molecule, and half a lattice water molecule (figure S2(a)). If the long Pb1–O31^iv^ (2.9358 (29) Å) is taken into consideration, the Pb1(II) ion is in a holodirected coordination sphere which is six-coordinate with six arsonate oxygen atoms deriving from six different ligands. The Pb2(II) ion is in a hemidirected coordination sphere that is completed by four arsonate oxygen atoms and one nitrogen atom that are provided by five different ligands. The Pb–O distances (2.236(2)–2.671(2) Å) are in the expected range. The Pb2–N31 distance (2.676(3) Å) is much shorter than the previously discussed bond in **1**. When only the relatively strong Pb–O bonds (Pb–O < 2.88 Å) are taken into account, the valence of Pb1 is 1.828. Further calculation demonstrates that the long Pb1–O31^iv^ bond has a considerable contribution, increasing the valence to 1.936. The valence of Pb2 calculated from the existing Pb–O bonds and the Pb2–N31 bond is 2.02, which is very close to the assumed oxidation state of the Pb(II) ion. The longest As–O bonds in the respective {AsO_3_} groups are As11–O13 1.742(3), As21–O23 1.699(2), and As31–O33 1.709(3) Å, so, considering the charge balance of **2**, O13 and O33 were assigned as hydroxyl atoms. L_As11_, L_As21_, and L_As31_ adopt *κ*O11-*μ*_1_, (*κ*O21**-***κ*O22, O22-*κ*O23, O23)-*μ*_5_, and (*κ*O31, O31**-***κ*O32, O32-*κ*N31)-*μ*_5_ coordination modes, respectively (figure 2(a)). The three different {AsO_3_} functionalities from L_As11_, L_As21_, and L_As31_ link the Pb(II) ions into 1D chains that extend in the direction of the crystallographic *b*-axis (figure 2(b)) and which are connected to each other by an L_As31_ spacer to form a 2D layer structure (figure 2(c)). There are two distinct, strong intramolecular hydrogen-bond interactions in **2** that occur within the 2D layer between OH functionalities (O13 and O33) of the AsO_3_ groups and deprotonated oxygen atoms (O23 and O12) of neighbouring AsO_3_ groups. These H-bonds are characterized by O13–H13···O23 and O33–H33···O12^vi^ distances of 2.649(4) and 2.493(4) Å, respectively [symmetry codes: (vi) *x*, *y* + 1, *z*] (figure S2(b)).

**[Pb**_**2**_**Cl**_**2**_**(TPAA)H**_**2**_**O]** (**3**) The asymmetric unit of **3** contains two Pb(II) ions, two terminal coordinated chloride anions, one fully deprotonated TPAA^2–^ ligand (denoted as L_As1_), and one *μ*_2_ bridging water molecule (figure S3). The compound crystallizes in the orthorhombic space group *Pccn*. Both two Pb(II) ions are in hemidirected coordination spheres. The Pb1(II) ion is six-coordinate with three arsonate oxygen atoms and one nitrogen atom deriving from three different ligands, one chlorine atom, and the *μ*_2_-bridging water molecule. The Pb2(II) ion is five-coordinate, whereby two arsonate oxygen atoms and one nitrogen atom are provided by three different ligands, one chlorine atom, and the *μ*_2_-coordinated water molecule, completing the distorted coordination sphere of this ion. The bond length of Pb2–O4W is 2.906(9) Å, which may be regarded as a semi-coordination mode. Except for Pb2–O4W, the other Pb–O distances are in the expected range, varying between 2.272(8) and 2.783 (9) Å. The distances of Pb1–N1^ii^ and Pb2–N2^ii^ are 2.737(10) and 2.851(9) Å, respectively, and those of Pb1–Cl1 and Pb2–Cl2 are 2.6855(30) and 2.7211(33) Å, respectively. The distance of Pb2–N2^ii^ is a little longer than the discussed Pb–N bond in **1** (2.837 (8) Å), whilst the Pb–Cl distances are in the expected range. The valence of Pb1 calculated from all the existing bonds (including the Pb2–O4W (2.906(9) Å) bond) is 2.07, which is very close to the assigned oxidation state of + II. If only the relatively strong Pb–O bonds (Pb–O < 2.88 Å) are taken into account, the valence of Pb2 is 1.95.

**Figure 3.**
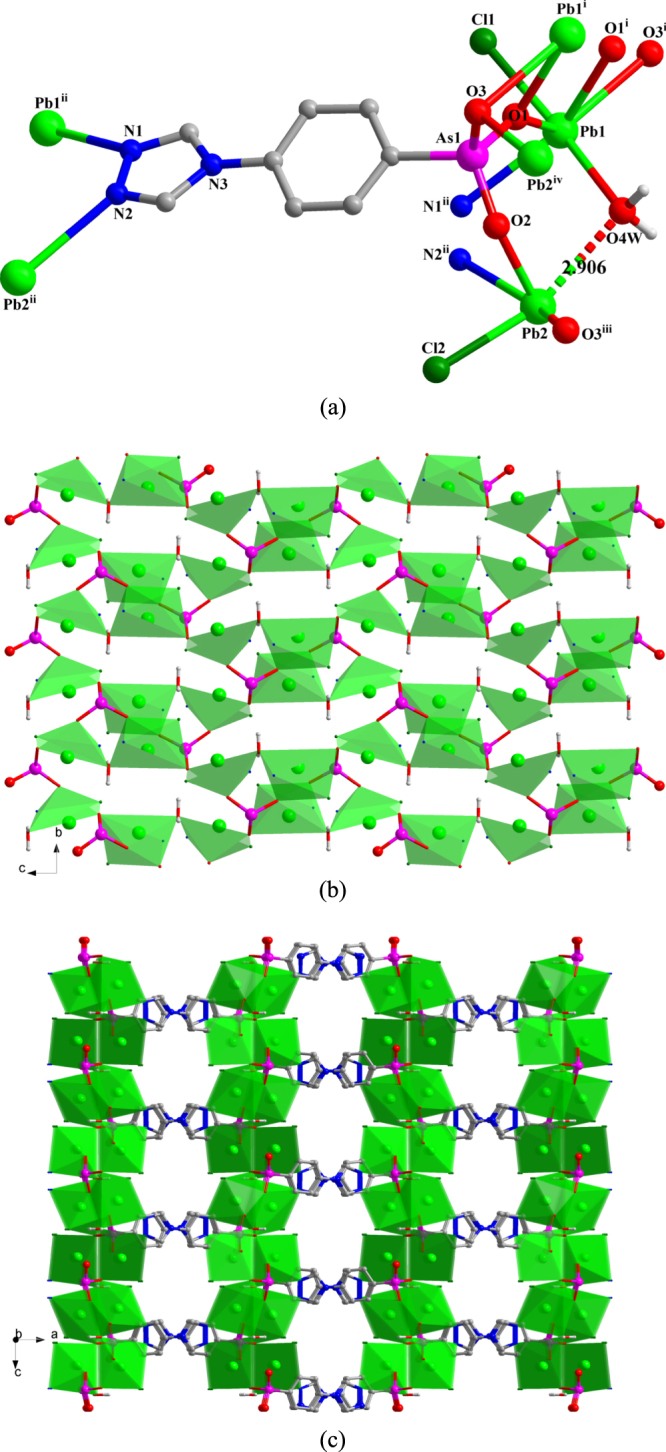
(a) The coordination environment of Pb(II) ions and the coordination mode of the ligand in **3**. Symmetry codes: (i) −*x* + 1, −*y* + 1, −*z* + 2; (ii) −*x* + 3/2, −*y* + 3/2, *z*; (iii) −*x* + 1, *y*−1/2, −*z* + 3/2; (iv) −*x* + 1, *y* + 1/2, −*z* + 3/2. (b) Polyhedral representation of two-dimensional layer structure in **3** comprising Pb(II) ions and the {As1O_3_} functionalities (view in the direction of the crystallographic *a*-axis). (c) Polyhedral representation of the three-dimensional framework of **3** (view in the direction of the crystallographic *b*-axis).

In **3**, the L_As1_ ligand adopts a (*κ*O1, O1**-***κ*O2-*κ*O3, O3-*κ*N1-*κ*N2)-*μ*_6_ coordination mode (figure 3(a)). The AsO_3_ functionalities from L_As1_ link Pb(II) ions into 2D layers that extend parallel to the *ac*-plane (figure 3(b)) and which are connected to each other by the L_As1_ spacer to form a 3D framework (figure 3(c)). Intramolecular hydrogen-bond interaction occurs in **3** between the coordinated water molecule and the {AsO_3_} group (O3) to give a O4W—H4WB···O3^v^ distance of 2.751(10) Å [Symmetry code: (v) *x*, *y*−1, *z*].

**[Pb**_**3**_**Cl(TPAA)(HTPAA)**_**2**_**H**_**2**_**O]Cl** (**4**) There are three Pb(II) ions, two HTPAA^–^ ligands, one fully deprotonated TPAA^2–^ ligand (denoted as L_As11_, L_As21_, and L_As31_), one terminal coordinated chlorine atom, one terminal coordinating water molecule, and one lattice chloride ion in the asymmetric unit of **4** (figure S4(a)). The compound crystallizes in the triclinic space group *P-*1. The Pb1(II)-Pb2(II) ions can be considered to adopt hemidirected coordination spheres. However, if the long Pb3–O22 bond (2.888(6) Å) is taken into consideration, the coordination environment of Pb3(II) may be described as holodirected. The Pb1(II) ion is five-coordinate with four arsonate oxygen atoms originating from three different ligands; the remaining binding site is occupied by a chloride ion. The Pb2(II) ion is five-coordinate, whereby four arsonate oxygen atoms and one nitrogen atom are provided by five different ligands. The six-coordinate binding environment of the Pb3(II) ion is composed of four oxygen and one nitrogen atom derived from five different ligands and one coordinated water molecule. The bond lengths of Pb–O range from 2.361(5) to 2.888(6) Å. The bond lengths of Pb2–N21^ii^ and Pb3–N31^iv^ are 2.521(7) and 2.737(7) Å, respectively, and that of Pb1–Cl41 is 2.753(2) Å. The valences of Pb1 and Pb2 calculated from all the existing bonds are 1.85 and 1.87, respectively, being smaller than the assigned oxidation state of +II. However, no other weak Pb–O interactions can be found around the Pb1(II) and Pb2(II) centers. If only the relatively strong Pb–O bonds (Pb–O < 2.88 Å) are taken into account, the valence of Pb3 is 1.88. Further calculation shows that the long Pb3–O22 interaction also makes a considerable contribution, increasing the valence to 2.004 for Pb3.

**Figure 4.**
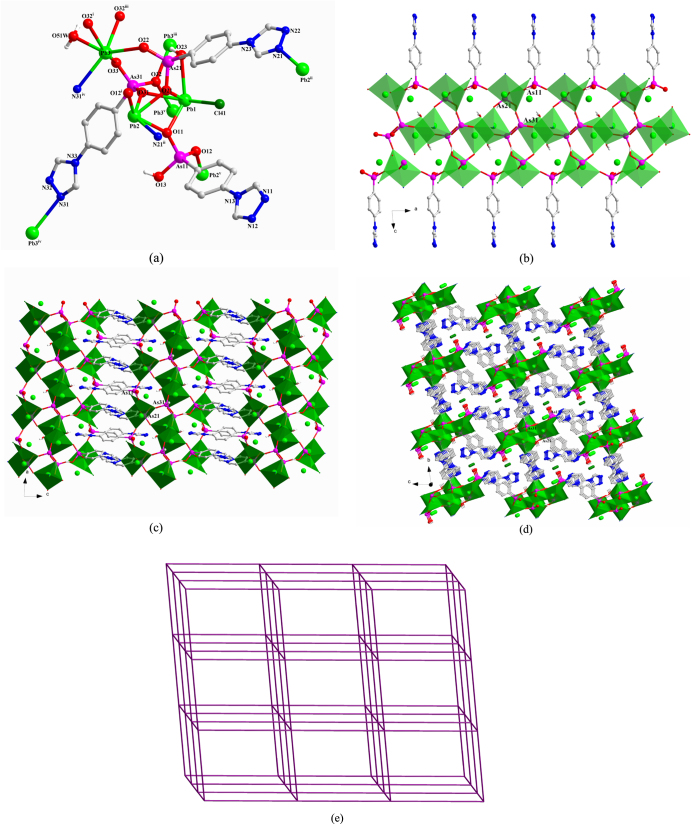
(a) The coordination environment of the Pb(II) ions and the coordination modes of the ligands in **4**. Symmetry codes: (i) *x*−1, *y*, *z*; (ii) −*x*, −*y*, −*z*; (iii) −*x*, −*y*, −*z* + 1; (iv) −*x*, −*y* + 1, −*z* + 1; (v) *x* + 1, *y*, *z*. (b) Polyhedral representation of the one-dimensional sub-structure in **4** involving Pb(II) ions and the three different {AsO_3_} functionalities (view in the direction of the crystallographic *b*-axis). (c) Polyhedral representation of the two-dimensional layer sub-structure in **4** in which L_As21_ moieties link between the adjacent one-dimensional subunits (view in the direction of the crystallographic *b*-axis). (d) Polyhedral representation of the three-dimensional framework structure in **4** in which L_As31_ moieties pillar between the adjacent two-dimensional layers (view in the direction of the crystallographic *a*-axis). (e) The NaCl-type network representing the topology of the 3D framework in **4**.

In **4**, L_As11_, L_As21_, and L_As31_ adopt (*κ*O11-*κ*O12)-*μ*_2_, (*κ*O21, O21**-***κ*O22-*κ*O23-*κ*N21)-*μ*_4_, and (*κ*O31, O31**-***κ*O32, O32-*κ*O33-*κ*N31)-*μ*_6_ coordination modes, respectively (figure 4(a)). The three different {AsO_3_} functionalities from L_As11_, L_As21_, and L_As31_ link the Pb(II) ions into 1D chains that extend in the direction of the crystallographic *b*-axis (figure 4(b)), and which are connected to each other by the L_As21_ spacer to form a 2D layer structure (figure 4(c)). These 2D layers are further linked by the L_As31_ spacer to form a 3D framework. The lattice chloride anions are located in the channels of the 3D framework (figure 4(d)). From the topological point of view, the resulting Pb_6_ clusters (two Pb1, two Pb2, and two Pb3, denoted as S) can be viewed as nodes. Connectors between adjacent S nodes are provided by L_As21_ and L_As31_ (17.480(4) and 13.708(3) Å) and through inter-node linkages within the 1D chain (7.2305(14) Å (see SI, figures S4(b) and (c))). Consequently, the 3D framework of **4** can be abstracted as a six-connected network with the Schläfli and vertex symbols being (4^12^6^3^) and 4·4·4·4·4·4·4·4·4·4·4·4·∗·∗·∗, respectively. This topology is consistent with a **pcu-**type network, according to the Reticular Chemistry Structure Resource (RCSR) notation (figure 4(e)). There are two distinct, strong intramolecular hydrogen-bond interactions in **4** within the 3D framework: one type of interaction occurs between OH functionalities (O23) of the {As21O_3_} groups and deprotonated oxygen atoms (O33) of neighbouring {As31O_3_} groups; the other type of interaction prevails between the coordinated water (O51W) and the neighbouring N11 atom. These H-bonds are characterized by O23–H23···O33^iii^ and O51W–H51A···N11^vi^ distances of 2.527(8) and 2.880(10) Å, respectively [symmetry codes: (iii) −*x*, −*y*, −*z* + 1; (vi) *x*−1, *y*, *z* + 1].

### 3.3. X-ray powder, FTIR spectral, and thermogravimetric analyses

The phase-purity of the here presented compounds was confirmed by PXRD. The 2 theta values of the major reflections of the experimentally recorded PXRD patterns of the bulk solids of **1**–**4** match well to those of the simulated patterns, which were calculated from respective single-crystal data (SI, figures S5(a)–(d)). The FTIR spectra of the four compounds show typical As–C stretching vibration bands at 1098 cm^−1^ for **1**, 1100 cm^−1^ for **2**, 1097 cm^−1^ for **3**, and 1099 cm^−1^ for **4**. The vibrations associated with the {AsO_3_} moiety are very strong and occur at 864 and 851 cm^−1^ for **1**, 873 cm^−1^ for **2**, 859 and 835 cm^−1^ for **3**, and 864 and 844 cm^−1^ for **4** (SI, figures S6(a)–(d)) [76]. The thermal stability of **1**–**4** was examined by TGA in an air atmosphere between 30–800 °C (SI, figure S7). The TGA curve of **1** reveals the removal of constitutional lattice water molecules and the coordinated water molecule in a range between 30–320 °C. **2** loses its lattice water and DMF molecules between 30–280 °C. After 280 °C, the framework of **2** undergoes oxidative degradation. The coordinated water molecule of **3** is lost between 30–110 °C, after which the framework architecture of **3** decomposes gradually due to the oxidation of the organic ligand. The coordinated water molecule of **4** is lost between 30–245 °C. The remaining structure of **4** is stable up to ∼305 °C, after which the framework collapses due to the combustion of the organoarsonate ligands.

### 3.4. Luminescence properties of 1–4

**Figure 5.**
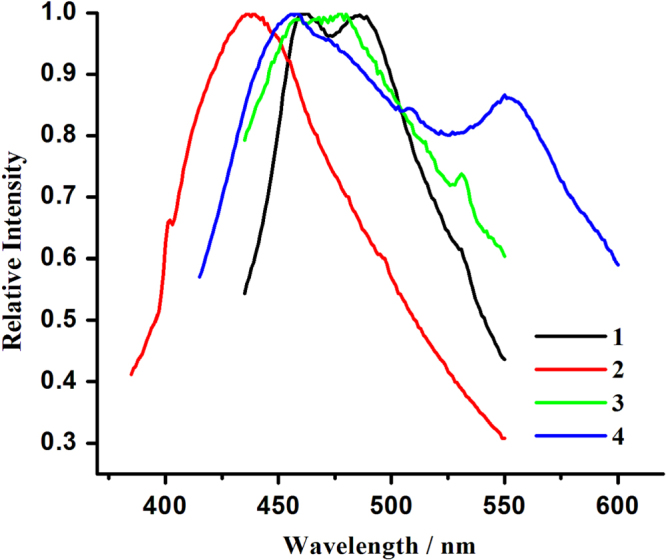
Emission spectra for **1**–**4** in the solid state at room temperature (excitation bands for **1**–**4** are 380 nm, 380 nm, 380 nm and 370 nm, respectively).

Hybrid coordination compounds containing Pb(II) ions may have interesting photochemical and photophysical properties [61–67, 86]. However, in comparison to many transition metal or lanthanide systems, the photoluminescence properties of lead(II)-organic frameworks are less explored. To further characterize **1**–**4**, their photoluminescence properties were investigated in the solid state at room temperature. As illustrated in figure 5, emission bands at 461 and 486 nm (*λ*_ex_ = 380 nm) for **1**, 438 nm (*λ*_ex_ = 380 nm) for **2**, 458, 478, and 531 nm (*λ*_ex_ = 380 nm) for **3** and 458 and 550 nm (*λ*_ex_ = 370 nm) for **4** are observed. For H_2_TPAA, an emission band maximum centered at 456 nm is apparent upon photoexcitation at 373 nm (SI, figure S8). The emission bands at 461 nm of **1**, 438 nm of **2**, 458 nm of **3**, and 458 nm of **4** may be due to the *π* → *π*∗ transition, as an approximate emission peak (456 nm) also appears in the spectra of the H_2_TPAA ligand. The emission bands at 486 nm of **1** and 478 nm of **3** can be attributed to ligand-to-metal charge transfer (LMCT) transitions involving delocalized *π* bonds of the aromatic arsonate groups and the *p* orbitals of Pb(II) centers. The low-energy emissions with large stokes shift, characteristic for the bands at 531 nm for **3** and 550 nm for **4**, can be assigned to metal-centered transitions involving *s* and *p* orbitals, as proposed by Vogler [87, 88].

## 4. Conclusions

In summary, four lead(II) coordination polymers with distinctively different structural motifs were successfully isolated using hydro(solvo)thermal preparative conditions. The applied preparative approach utilized different lead reactants that were reacted with various metal/ligand molar ratios with a bifunctional organoarsonate ligand in H_2_O or DMF. These applied reaction parameters, including the counterions, played a crucial role of the topology and composition of the resulting Pb(II) coordination polymers. The results demonstrate that the coordination modes of the H_2_TPAA ligand are highly flexible, adopting *μ*_1_*−*, *μ*_2_*−*, *μ*_3_*−*, *μ*_4_*−*, *μ*_5_*−*, and *μ*_6_*−* bridging modes. **1** and **2** represent 2D layered structures, whilst **3** and **4** form 3D frameworks. The structures of **1**, **2**, and **4** contain 1D {Pb-AsO_3_} substructures, while the framework of **3** is characterized by 2D {Pb-AsO_3_} sub-structural motifs. Compounds **1**–**4** all exhibit photoluminescence properties in the solid state at room temperature. The arsonic acid functionalities in the examined compounds have a high propensity to be partially protonated under the applied reaction conditions. Among these four lead(II) coordination polymers, only **3** is stabilized by fully deprotonated arsonate functionalities. This reaction behaviour and the consequent lower tendency to bridge metal centers than corresponding phosphonate ligands can be interpreted in light of their p*K*_a_ values. According to the literature, the p*K*_a_ values of phenylphosphonic acid are 1.86 and 7.51 [89], and the p*K*_a_ values of phenylarsonic acid are 3.8 and 8.5 [90], suggesting that the 2nd deprotonation event occurs more readily for phosphonic acids than that for the arsonic acids. Despite the fact that arsonate anions can also adopt a variety of potential coordination modes, their ability as complexing agents has not yet been fully explored. Consequently, new synthetic methodologies are required to explore to further fully understand the coordination chemistry of organoarsonates.
